# Malvidin’s Effects on Rat Pial Microvascular Permeability Changes Due to Hypoperfusion and Reperfusion Injury

**DOI:** 10.3389/fncel.2016.00153

**Published:** 2016-06-30

**Authors:** Dominga Lapi, Martina Chiurazzi, Martina Di Maro, Teresa Mastantuono, Laura Battiloro, Lina Sabatino, Serena Ricci, Angelina Di Carlo, Noemy Starita, Bruna Guida, Mariarosaria Santillo, Antonio Colantuoni

**Affiliations:** ^1^Department of Clinical Medicine and Surgery, School of Medicine, University of Naples Federico IINaples, Italy; ^2^Department of Science and Technology, University of SannioBenevento, Italy; ^3^Department of Translational Medicine, University of Naples Federico IINaples, Italy; ^4^Department of Medico-Surgical Sciences and Biotechnologies, Sapienza University of RomeRome, Italy

**Keywords:** bilateral common carotid artery occlusion, reperfusion, pial microcirculation, endothelial nitric oxide synthase, metalloproteinases, zymography, radical oxygen species, malvidin

## Abstract

The present study was aimed to evaluate the malvidin’s protective effects on damage induced by 30 min bilateral common carotid artery occlusion (BCCAO) and 60 min reperfusion (RE) in rat pial microcirculation. Rat pial microcirculation was observed using fluorescence microscopy through a closed cranial window. Western blotting analysis was performed to investigate the endothelial nitric oxide synthase (eNOS), phosphorylated eNOS (p-eNOS) and matrix metalloproteinase 9 (MMP-9) expression. Moreover, MMP-9 activity was evaluated by zymography. Finally, neuronal damage and radical oxygen species (ROS) formation were assessed. In all animals, pial arterioles were classified in five orders of branching according to Strahler’s method. In hypoperfused rats, 30 min BCCAO and 60 min RE caused a decrease in arteriolar diameter, an increase in microvascular leakage and leukocyte adhesion, accompanied by decreased capillary perfusion and red blood cell velocity (V_RBC_). Moreover, marked neuronal damage and evident ROS generation were detected. Conversely, malvidin administration induced arteriolar dilation in dose-related manner, reducing microvascular leakage as well as leukocyte adhesion. Capillary perfusion and V_RBC_ were protected. Nitric oxide (NO) synthase inhibition significantly attenuated malvidin’s effects on arteriolar diameter. Western blotting analysis revealed an increase in eNOS and p-eNOS expression, while zymography indicated a decrease in MMP-9 activity after malvidin’s administration. Furthermore, malvidin was able to prevent neuronal damage and to decrease ROS generation. In conclusion, malvidin protects rat pial microcirculation against BCCAO/RE injury, preventing blood-brain impairment and neuronal loss. Malvidin’s effects appear to be mediated by eNOS activation and scavenger activity.

## Introduction

Several studies indicate that pial microvascular changes during hypoperfusion and reperfusion (RE) injury can mimic the alterations in cerebral microcirculation during ischemic insults (Lapi et al., 2008b, 2015). Pial arterioles, indeed, are the main site of blood flow control to the superficial layer of cerebral cortex, while pial venules drain most of cerebral cortex blood flow. In particular, haemodinamic changes in pial microvascular network trigger complex mechanisms involving blood brain barrier (BBB) integrity and consequent influence on neurons and astrocytes (Iadecola, 2004; Lapi et al., 2015). The experimental model of bilateral common carotid artery occlusion (BCCAO) and RE permits to study the early microvascular responses of pial vessels leading to the BBB disruption. Previous data have shown that brain hypoperfusion, induced by BCCAO, and subsequent RE cause significant damages in pial microcirculation, characterized by vasoconstriction, BBB impairment, leukocytes adhesion, reduction in capillary perfusion and consequent neuronal loss (Ywasaki et al., 1989 ; Yanpallewar et al., 2004). In particular, BCCAO determines radical oxygen species (ROS)/nitric oxide (NO) imbalance inducing oxidative stress with subsequent reduced arteriolar diameter and venular wall alterations. These changes are accompanied by interstitial edema as indicated by leakage of fluorescent tracers (Lapi et al., 2012, 2015; Mastantuono et al., 2015). Furthermore, vascular injury during brain ischemia may be due to activation of matrix metalloproteinase-9 (MMP-9). It is well known that MMPs are secreted into the extracellular space, as inactive zymogens, and successively activated; in particular, MMP-9 appears to be activated during ischemia. Moreover, some pro-inflammatory cytokines (TNFα and IL-1β) as well as ROS have been proven to trigger a significant increase in MMP-9 expression (Lehmann et al., 2005; Hsieh and Yang, 2013). Therefore, ischemia-activated MMPs could degrade the extracellular matrix, leading to the opening of the BBB (Fujimura et al., 1999; Aoki et al., 2002; Candelario-Jalil et al., 2009).

Our previous data, moreover, have shown that antioxidants, widely present in nature, are effective in counteracting the vessel wall damage, reducing microvascular leakage as well as leukocyte adhesion along pial venular walls, thus facilitating tissue perfusion (Lapi et al., 2015; Mastantuono et al., 2015). Many studies have reported the antioxidant and anti-inflammatory properties of antocyanins present in berries and grapes, indicating they could be useful in many pathophysiological conditions. Among these molecules, malvidin 3-O- glucoside appears to be one of the most abundant (Quintieri et al., 2013).

Up to day, no data have been reported on the protective effects of malvidin against cerebral injury, due to hypoperfusion and RE. Therefore, the aim of the present study was to evaluate the protective effects of malvidin on damage induced by BCCAO and RE in the rat pial microcirculation, using *in vivo* fluorescence microscopy.

## Materials and Methods

### Experimental Groups

Male Wistar rats, weighing 250–300 g (Harlan, Italy), were randomly divided into two groups: sham-operated group (S group) and hypoperfused group (Hypo group).

The animals of the S group, submitted to the same surgical procedures as the other experimental group without BCCAO and RE, were differentiated in three subgroups: (1) Sham-saline subgroup (*n* = 14) received intravenous (i.v.) saline solution (0.9% NaCl); (2) Sham-M_2_ subgroup (*n* = 5) was treated with i.v. higher dosage malvidin, 18 mg/kg body weight (b.w.); (3) Sham-L subgroup (*n* = 5) was administered with i.v. L-NIO, 10 mg/kg b.w. All substances were administered twice at 40 min time-interval.

Hypoperfused group, subjected to 30 min BCCAO and 60 min RE, was divided in the following subgroups: (1) Hypo subgroup (*n* = 14) was treated with i.v. saline solution (0.9% NaCl), injected 10 min before BCCAO and at the beginning of RE; (2) Hypo-M_1_ and Hypo-M_2_ subgroups (*n* = 5 and *n* = 14, respectively) received i.v. malvidin, 9 or 18 mg/kg b.w., respectively, 10 min before BCCAO and at the beginning of RE; (3) Hypo-L/M_2_ subgroup (*n* = 14) was treated with i.v. L-NIO 10 mg/kg b.w., prior to i.v. higher dosage malvidin (18 mg/Kg b.w.).

In Sham-saline, Hypo, Hypo-M_2_ and Hypo-L/M_2_ subgroups five animals were used for microvascular studies, three rats were utilized for western blotting analysis and zimography, three animals were used to determine neuronal damage by triphenyl tetrazolium chloride (TTC) staining and three rats were submitted to 2’-7’-dichlorofluorescein-diacetate (DCFH-DA) assay after RE. The remaining subgroups were submitted only to microvascular studies.

### Administration of Drugs

Malvidin solution was obtained dissolving 9 or 18 mg/Kg b.w., in 0.5 mL saline solution and i.v. infused (3 min) 10 min before BCCAO and at the beginning of RE. In pilot experiments malvidin was tested in different concentrations to choose the dosages useful for the present study. Malvidin concentrations less than 9 mg/kg b.w. did not exert significant effect on the pial microcirculation, while doses above 18 mg/kg b.w. did not significantly increase the protective effects observed in the rats treated with malvidin at the dose of 18 mg/kg b.w. and subjected to hypoperfusion and RE.

L-NIO (10 mg/kg b.w.) was dissolved in 0.5 mL saline solution and i.v. administered 10 min prior to i.v. higher dosage malvidin (18 mg/kg b.w.), 10 min before BCCAO and at the beginning of RE. Pilot experiments indicated that the dosages of L-NIO utilized in the present study abolished dilation of rat pial arterioles caused by intravenous infusion of L-arginine, 10 mg/4 min (diameter increase by 23 ± 3% of baseline) or blunted vasodilation due to topical application of acetylcholine, 100 μM (diameter increase by 4 ± 1% of baseline).

Drugs were administered according to the protocol previously reported (Lapi et al., 2015). DCFH-DA was mixed with artificial cerebrospinal fluid (aCSF) to obtain a concentration of 250 mM (Watanabe, 1998). This solution was superfused over the pial surface for 30 min at the beginning of RE. All drugs were purchased from Sigma Chemical, St. Louis, MO, USA.

### Animal Preparation

All experiments conform to the Guide for the Care and Use of Laboratory Animals published by the USA National Institutes of Health (NIH Publication No. 85–23, revised 1996) and to institutional rules for the care and handling of experimental animals. The protocol was approved by the “Federico II” University of Naples Ethical Committee.

Rats were anesthetized with an initial intraperitoneal injection (i.p.) of α-chloralose (60 mg/kg b.w.) and maintained by repeated intravenous injections (i.v.) of α-chloralose (30 mg/kg b.w. every hour). Animals were paralyzed with tubocurarine chloride (1 mg/kg.h, i.v.), tracheotomized and mechanically ventilated with room air and supplemental oxygen. The right and left common carotid arteries were isolated for successive clamping. A catheter was placed in the left femoral artery to measure arterial blood pressure; the one in the right femoral vein for drug and fluorescent tracer injection [fluorescein isothiocyanate bound to dextran, molecular weight 70 kDa (FD 70), 50 mg/100 g b.w., as 5% wt/vol solution in 3 min administered just once at the beginning of experiment after 30 min of the preparation stabilization; rhodamine 6G, 1 mg/100 g b.w. in 0.3 mL, as a bolus with supplemental injection throughout BCCAO and RE (final volume 0.3 mL·100 g−1·h−1) to label leukocytes for adhesion evaluation]. Blood gas measurements were carried out on arterial blood samples withdrawn from arterial catheter at 30 min time period intervals (ABL5; Radiometer, Copenhagen, Denmark). Throughout all experiments, mean arterial blood pressure, heart rate, respiratory CO_2_ and blood gases values were recorded and stable settled within physiological ranges. Rectal temperature was monitored and preserved at 37.0 ± 0.5°C, as previously reported (Lapi et al., 2015).

To observe the pial microcirculation, a closed cranial window (4 mm × 5 mm) was implanted above the left frontoparietal cortex (posterior 1.5 mm to bregma; lateral, 3 mm to the midline, according to the method previously described (Ngai et al., 1988). Briefly, a 1 cm incision was made in the skin to expose the skull and a craniotomy was performed. Cold saline solution was suffused on the skull during drilling to avoid overheating of cerebral cortex. The dura mater was gently removed and a 150-μm-thick quarz microscope coverglass was sealed to the bone with dental cement. The brain parenchyma was continuously superfused with artificial cerebrospinal fluid (aCSF; Hudetz et al., 1985; Morii et al., 1986). The rate of superfusion was 0.5 mL/min controlled by a peristaltic pump. The composition of the aCSF was 119.0 mM NaCl, 2.5 mM KCl, 1.3 mM mgSO_4_·7H_2_O, 1.0 mM NaH_2_PO_4_, 26.2 mM NaHCO_3_, 2.5 mM CaCl_2_ and 11.0 mM glucose (equilibrated with 10.0% O_2_, 6.0% CO_2_ and 84.0% N_2_; pH 7.38 ± 0.02). The temperature was maintained at 37.0 ± 0.5°C with a water bath.

Two atraumatic microvascular clips were placed on common carotid arteries, previously isolated, to obtain the hypoperfusion. After 30 min, they were removed to observe the pial microcirculation in the RE period (60 min).

### Fluorescence Intravital Microscopy

Pial vessels were observed with a fluorescent microscope (Leitz Orthoplan, Wetzlar, Germany) fitted with long-distance objectives (2.5×, numerical aperture (NA) 0.08; 10×, NA 0.20; 20×, NA 0.25; 32×, NA 0.40) a 10× eyepiece and a filter block (Ploemopak, Leitz). Epiillumination was provided by a 100-Watt mercury lamp using the appropriate filters for FITC, for rhodamine 6G, and a heat filter (Leitz KG1). The pial microcirculation was televised with a DAGE MTI 300 low-light level digital camera and recorded by a computer based frame grabber (Pinnacle DC 10 plus, Avid Technology, Burlingtton, MA, USA).

### Geometric Analysis of Arteriolar Network

Under baseline conditions, the arteriolar network was mapped by stop-frame images and pial arterioles were classified according to a centripetal ordering scheme (Strahler’s method, modified according to diameter; Kassab et al., 1993; Lapi et al., 2008a). Order 0 was assigned to the capillaries; thereafter, the terminal arterioles were assigned order 1 and the vessels upstream were assigned progressively higher order. When two vessels of the same order joined, the parent vessel was assigned the next highest order. If two daughter vessels were of different orders, the parent vessel retained the higher of the two orders. In each animal one order 4 arteriole, two order 3, and two order 2 arterioles were studied during each experiment. We studied the responses of each arteriolar order to the experimental conditions; however, we chose to present the data about order 3 vessels.

### Microvascular Parameter Evaluation

Microvascular measurements were made off-line using a computer-assisted imaging software system (MIP Image, CNR, Institute of Clinical Physiology, Pisa, Italy). Recording of microvascular images was performed for 1 min every 5 min during baseline, before BCCAO and at the beginning of RE. Afterwards, recording was carried out every 10 min during BCCAO and the remaining RE. The baseline conditions were represented by microvascular values detected within 2 min of FITC administration.

Arteriolar diameters and capillary red blood cell velocity (V_RBC_) were measured with a computer-assisted method (MIP Image program, frame by frame). The results of diameter measurements were in accord with those obtained by shearing method (±0.5 μM). To avoid bias due to single operator measurements, two independent “blinded” operators measured the vessel diameters. Their measurements overlapped in all cases.

The increase in permeability was calculated and reported as normalized gray levels (NGL): NGL = (I−Ir)/Ir, where, Ir is the average baseline gray level at the end of vessel filling with fluorescence (average of 5 windows located outside the blood vessels with the same windows being used throughout the experimental procedure), and I is the same parameter at the end of BCCAO or at the end of RE. Gray levels ranging from 0 to 255 were determined by the MIP Image program in five regions of interest (ROI) measuring 50 × 50 μM (10× objective). The same location of ROI during recordings along the microvascular networks was provided by a computer-assisted device for *XY* movement of the microscope table.

Adherent leukocytes (i.e., cells on vessel walls that did not move over a 30-s observation period) were quantified in terms of number/100 μM of venular length (v.l.)/30 s using higher magnification (20× and 32×, microscope objectives). In each experimental group 45 venules were studied.

Perfused capillary length (PCL) was measured by MIP image in an area of 150 × 150 μM. In this system the length of perfused capillaries is easily established by the automated process because it is outlined by dextran (Colantuoni et al., 2005).

Mean arterial blood pressure (Viggo-Spectramed P10E2 transducer; Oxnard, CA, USA—connected to a catheter in the femoral artery) and heart rate were monitored with a Gould Windograf recorder (model 13–6615–10S, Gould, OH, USA). Data were recorded and stored in a computer. Blood gas measurements were carried out on arterial blood samples withdrawn from arterial catheter at 30 min time period intervals (ABL5; Radiometer, Copenhagen, Denmark). The hematocrit was measured under baseline conditions, at the end of BCCAO and at the end of RE.

### Western Blotting Analysis

Cortex and striatum tissues were homogenized in a Polytron (Brinkman Instruments, NY, USA) in lysis buffer containing 50 mM HEPES, 150 mM NaCl, 5 mM EGTA, 150 mM MgCl_2_, 1% glycerol, 1% Triton X-100, 1 mM PMSF, 1 mM trypsin inhibitor. Protein concentration was quantified by the Bradford assay (Bio-Rad, Berkeley, CA, USA). The homogenate was stirred for 1 h at 4°C and then centrifuged at 14,000 rpm × 2′min. The supernatant was collected and protein determined by the Bradford procedure (Bio-Rad, Berkeley, CA, USA). Equal amounts of proteins were run on 7.5% SDS-PAGE under reducing conditions, then transferred to polyvinylidene difluoride membranes (PVDF; Invitrogen, Carlsbad, CA, USA). The membrane was blocked for 1 h with 5% w/v BSA in Tris-buffered saline and 0.1% Tween 20 (TBST) at room temperature. Filters were incubated with specific antibodies at 4°C overnight, washed in TBST and then incubated with horseradish peroxidase-conjugated secondary antibody (1:1000; GE-Healthcare, Little Chalfont, UK) for 1 h at room temperature. After washing, peroxidase activity was detected by ECL system (GE-Healthcare, Little Chalfont, UK). Protein loading was normalized by incubating the same filters with anti-β-actin antibody (Sigma Aldrich; Milan, Italy) and the band intensity was quantified by densitometry (ChemiDoc XRS, Bio-Rad). We evaluated the protein concentration of endothelial NO synthase (eNOS) and p/eNOS compared to β/actin concentration and calculated the ratio p/eNOS/eNOS concentrations.

To detect the proteins of interest, specific antibodies were utilized: rabbit polyclonal anti-eNOS (1:1000), rabbit polyclonal anti-phosphorylated eNOS (Ser1177) (1:1000) and anti-MMP-9 (1:500). Anti-eNOS, anti-p-eNOS and anti-β-actin antibodies were purchased from Cell Signaling Technology Inc., (Danvers, MA, USA); while anti-MMP-9 antibody from Sigma-Aldrich (St. Louis, MO, USA).

### Gelatin Zymography

Equal amounts of proteins from each sample homogenate were mixed with sample buffer (10 mM Tris-HCl pH 6.8, 12.5% SDS, 5% sucrose, 0.1% bromophenol blue) and subjected to SDS-PAGE in 7.5% polyacrylamide gels, containing 0.1% (w/v) of gelatine. After removal of SDS from the gels by incubation in 2.5% (w/v) Triton X-100 for 1 h, the gels were incubated at 37°C for 18 h in 50 mM Tris-HCl pH 7.6 containing 0.2 M NaCl, 5 mM CaCl_2_ and 0.02% (w/v). Gels were stained in 30% methanol, 10% glacial acetic acid containing 0.5% (w/v) Coomassie Brilliant Blue G 250 for 1 h and destained in the same solution without dye for several hours. The gelatinolytic activity of each collagenase was evident as a clear band against the blue background of stained gelatine. The molecular size of bands displaying enzymatic activity were identified by comparison with prestained standard protein, as well as with purified gelatinase A or gelatinase B.

### TTC Staining

Rats were sacrificed after 30 min BCCAO and 60 min RE. Tissue damage was evaluated by TTC staining. The brains were cut into 1 mM coronal slices with a vibratome (Campden Instrument, 752 M; Lafayette, IN, USA). Sections were incubated in 2% TTC for 20 min at 37°C and in 10% formalin overnight. The necrotic area site and extent in each section were evaluated by image analysis software (Image-Pro Plus; Rockville, MD, USA; Bederson et al., 1986).

### DCFH-DA Assay

Artificial cerebrospinal fluid containing 250 mM DCFH-DA at 37.0 ± 0.5°C was superfused over the pial surface. DCFH-DA is widely used as a marker for oxidative stress of the cell and tissue (Wang and Joseph, 1999). This hydrophobic non-fluorescent molecule penetrates rapidly into the cell, where it is hydrolyzed to DCFH by intracellular esterases. Successively, DCFH was oxidized to its fluorescent product (DCF) in the presence of ROS. The intensity of DCF fluorescence is proportional to the intracellular ROS level. The fluorescence intensity was determined by the use of an appropriate filter (522 nm) and estimated by NGL, comparing the DCF fluorescence at the end of RE with the baseline represented by pial surface just superfused by DCFH-DA (Watanabe, 1998).

### Statistical Analysis

All data were expressed as Mean ± SEM. Data were tested for normal distribution with the Kolmogorov-Smirnov test. Parametric (Student’s *t* tests, ANOVA and Bonferroni *post hoc* test) or nonparametric tests (Wilcoxon, Mann-Whitney and Kruskal-Wallis tests) were used; nonparametric tests were applied to compare diameter and length data among experimental groups. Due to the small sample size of DCFH-DA treated rats we used non-parametric tests to compare the results obtained in these animals. The statistical analysis was carried out by SPSS 14.0 statistical package (IBM Italia, Segrate, Italy). Statistical significance was set at *p* < 0.05.

## Results

Under baseline conditions, pial microvascular networks of all animals were characterized by Strahler’s centripetal ordering scheme, as previously reported (Lapi et al., 2008b): arterioles were classified from the largest vessels, assigned order 5 (average diameter 63.7 ± 4.7 μm), to the smallest ones, assigned order 1 (average diameter: 16.4 ± 2.3 μm). Capillaries, sprouting from order 1 arterioles, were assigned order 0.

**Figure 1:**
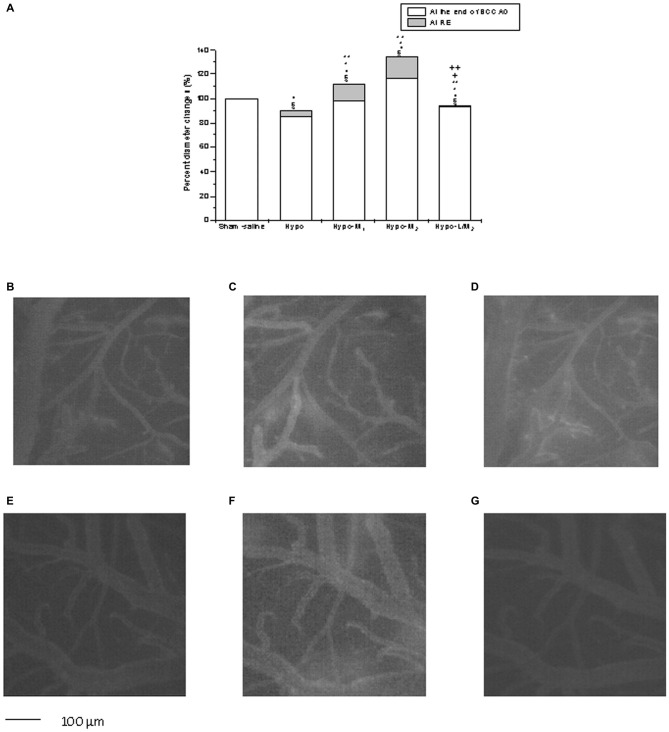
**Diameter changes in the experimental subgroups (A).** Diameter changes of order 3 arterioles, expressed in percentage of baseline at the end of bilateral common carotid artery occlusion (BCCAO) and reperfusion (RE) in Sham-saline = sham operated subgroup; Hypo = hypoperfused subgroup; Hypo-M_1_ = malvidin subgroup treated with lower dosage (9 mg/kg b.w.); Hypo-M_2_ = malvidin subgroup treated with higher dosage (18 mg/kg b.w.); Hypo-L/M_2_ = L-NIO (10 mg/kg b.w.) and higher dosage malvidin (18 mg/kg b.w.) subgroup. Data are reported as Mean ± SEM; ^§^*p* < 0.01 vs. Baseline; °*p* < 0.01 vs. Sham-saline subgroup; *p < 0.01 vs. Hypo subgroup at the end of BCCAO; **p < 0.01 vs. Hypo subgroup at the end of RE; ^+^*p* < 0.01 vs. Hypo-M_2_ subgroup at the end of BCCAO; ^++^*p* < 0.01 vs. Hypo-M_2_ subgroup at the end of RE. Computer-assisted images of a pial microvascular network under baseline conditions **(B)**, at the end of BCCAO **(C)** and RE **(D)** in one of the hypoperfused rats. The increase in microvascular leakage is outlined by the marked change in the color of interstitium (from black to white). Computer-assisted images of a pial microvascular network under baseline conditions **(E)** at the end of BCCAO **(F)** and RE **(G)** in a higher dosage malvidin-treated rat (18 mg/kg b.w.), where there was nitric oxide (NO) leakage of fluorescent-dextran.

During the entire observation period, Sham-saline subgroup did not show changes in arteriolar diameter, nor microvascular leakage (0.03 ± 0.01 NGL), nor leukocyte adhesion (0.5 ± 0.3/100 μm of venular length, v.l./30 s). All capillaries were perfused and V_RBC_ was 0.20 ± 0.02 mM/s (Table 1, Figure 1B).

**Table 1:** **Variations of the main parameters at the end of reperfusion in sham-operated (Sham-saline) subgroup; hypoperfused (Hypo) subgroup; malvidin (Hypo-M_1_) subgroup (9 mg/kg b.w.); higher dose malvidin (Hypo-M_2_) subgroup (18 mg/kg b.w.); L-NIO (10 mg/kg b.w) and malvidin (18 mg/kg b.w.) subgroup**.

Subgroups	Number of animals (n)	Microvascular leakage (NGL)	Leukocyte adhesion (number of leukocyte/100 μm of venular length/30 s)	Capillary perfusion (PCL) % reduction (compared to baseline)	Capillary red blood cell velocity (V_RBC_ mm/s)
Sham-saline	5	0.03 ± 0.01	0.5 ± 0.3	0 ± 4	0.20 ± 0.02
Hypo	5	0.46 ± 0.04^§^°	10 ± 2^§^°	48 ± 4^§^°	0.12 ± 0.03^§^°
Hypo-M_1_	5	0.32 ± 0.03^§^°*	6 ± 1^§^°*	35 ± 3^§^°*	0.17 ± 0.01^§^°*
Hypo-M_2_	5	0.10 ± 0.02^§^°*	2 ± 1*	28 ± 4^§^°*	0.19 ± 0.02*
Hypo-L/M_2_	5	0.13 ± 0.03^§^°*+	3.0 ± 1.5*	30 ± 2^§^°*+	0.24 ± 0.02*

For capillary perfusion, data at the end of reperfusion were compared with those prior to hypoperfusion. Leukocyte adhesion: n = 45 venules for each entry. Data are reported as Mean ± SEM; ^§^p < 0.01 vs. Baseline; °p < 0.01 vs. Sham-saline subgroup, *p < 0.01 vs. Hypo subgroup, ^+^p < 0.01 vs. Hypo-M_2_ subgroup.

In Sham-M_2_ subgroup, malvidin treatment determined a dose-related dilation of all arterioles; order 3 arterioles (average mean diameter: 34.2 ± 3.2 μm) dilated by 4.5 ± 0.4% of baseline with higher dosage malvidin. After drug treatment, there were no significant changes in the other parameters when compared to baseline. Conversely, L-NIO administration caused no significant changes in all parameters.

### Hypoperfusion and Reperfusion

BCCAO caused severe injuries in the pial microvascular networks. The animals (Hypo subgroup) showed a decrease in diameter of all arteriolar orders after 30 min BCCAO: the reduction was by 15.0 ± 2.5% of baseline in order 3 vessels (*p* < 0.01 vs. baseline and Sham-saline subgroup; Figure 1A). Microvascular leakage significantly increased compared with baseline (0.32 ± 0.04 NGL; *p* < 0.01 vs. baseline and Sham-saline subgroup) (Figure 1C).

At RE, order 3 arteriole diameter (average mean diameter: 33.9 ± 2.7 μm) decreased by 9.6 ± 1.5% of baseline (*p* < 0.01 vs. baseline and Sham-saline subgroup; Figure 1A), while microvascular leakage increased (0.46 ± 0.04 NGL; *p* < 0.01 vs. baseline and Sham-saline subgroup). Moreover, the number of leukocytes adhering to venular walls increased (10 ± 2/100 μm v.l./30 s; *p* < 0.01 vs. baseline and Sham-saline subgroup); PCL decreased by 48 ± 4% of baseline and V_RBC_ was 0.12 ± 0.03 mm/s (*p* < 0.01 vs. baseline and Sham-saline subgroup; Table 1, Figure 1D).

Conversely, Hypo-M1 subgroup, treated with lower dosage malvidin (9 mg/kg b.w.), did not show significant change in arteriolar diameter compared to baseline after 30 min BCCAO (*p* < 0.01 vs. Hypo subgroup; Figure 1A), while fluorescent spots along venular walls were significantly reduced compared to animals of Hypo subgroup (0.18 ± 0.02 NGL; *p* < 0.01 vs. baseline, Sham-saline and Hypo subgroups).

At RE, Hypo-M_1_ subgroup showed a significant increase in arteriolar diameter: order 3 arterioles (average mean diameter: 33.7 ± 2.9 μm) dilated by 11.9 ± 1.8% of baseline (*p* < 0.01 vs. baseline, Sham-saline and Hypo subgroups; Figure 1A). Furthermore, microvascular permeability significantly decreased compared to animals of Hypo subgroup (0.32 ± 0.03 NGL; *p* < 0.01 vs. baseline, Sham-saline and Hypo subgroups) as well as leukocyte adhesion (6 ± 1/100 μm v.l./30 s; *p* < 0.01 vs. baseline, Sham-saline and Hypo subgroups). Finally, PCL diminished by 35 ± 3% of baseline and V_RBC_ was 0.17 ± 0.01mm/s (*p* < 0.01 vs. baseline, Sham-saline and Hypo subgroups; Table 1).

Hypo-M_2_ subgroup received higher dosage malvidin (18 mg/kg b.w.) and revealed a significant dilation in arteriolar diameter: order 3 arterioles (average mean diameter: 33.8 ± 2.8 μm) dilated by 16.5 ± 2.9% of baseline at the end of BCCAO (*p* < 0.01 vs. baseline, Sham-saline and Hypo subgroups; Figure 1A). Microvascular permeability did not change compared to baseline (0.05 ± 0.01 NGL; *p* < 0.01 vs. Hypo subgroup).

At RE, the arteriolar diameter dilation was by 34.1 ± 3.2% of baseline (*p* < 0.01 vs. baseline, Sham-saline and Hypo subgroups; Figures 1A–G); moreover, microvascular leakage was slightly increased (0.10 ± 0.02 NGL; *p* < 0.01 vs. baseline, Sham-saline and Hypo subgroups) as well as leukocyte adhesion (2 ± 1/100 μm v.l./30 s; *p* < 0.01 vs. Hypo subgroup). Finally, PCL decreased by 28 ± 4% of baseline (*p* < 0.01 vs. baseline, Sham-saline and Hypo subgroups) and V_RBC_ was 0.19 ± 0.02 mm/s (*p* < 0.01 vs. Hypo subgroup; Table 1).

Hypo-L/M_2_ subgroup, treated with L-NIO (10 mg/kg b.w.) prior to the higher dosage malvidin administration, showed a significant reduction in diameters of all arteriolar orders compared to Hypo-M_2_ subgroup at the end of BCCAO: order 3 arteriolar diameter (average mean diameter: 33.9 ± 3.1 μm) decreased by 6.4 ± 1.2% of baseline (*p* < 0.01 vs. Hypo-M_2_ subgroup; Figure 1A). Microvascular permeability did not significantly change compared to Hypo-M_2_ subgroup (0.10 ± 0.02 NGL; *p* < 0.01 vs. Hypo subgroup).

At RE, order 3 arteriole diameter was reduced by 5.5 ± 1.2% of baseline (*p* < 0.01 vs. Hypo-M_2_ subgroup; Figure 1A); in addition, fluorescent leakage was slightly increased (0.13 ± 0.03 NGL; *p* < 0.01 vs. baseline, Sham-saline and Hypo subgroups) as well as leukocyte adhesion (3.0 ± 1.5/100 μm v.l./30 s; *p* < 0.01 vs. Hypo subgroup). Finally, PCL was reduced by 30 ± 2% of baseline (*p* < 0.01 vs. baseline, Sham-saline and Hypo subgroups) and V_RBC_ was 0.24 ± 0.02 mM/s (*p* < 0.01 vs. Hypo subgroup; Table 1).

### eNOS and MMP-9 Expressions

**Figure 2:**
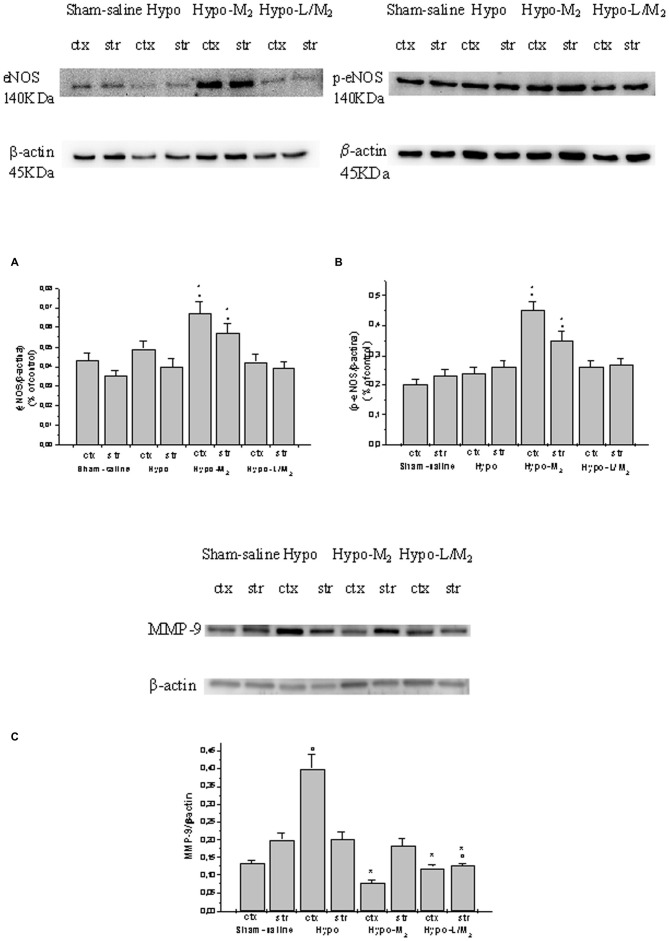
**Western blotting of endothelial NO synthase (eNOS; A), phosphorylated eNOS (B) and MMP-9 (C) expression in two cerebral zones, cortex (ctx) and striatum (str), at the end of RE in Sham-saline = sham operated subgroup; Hypo = hypoperfused subgroup; Hypo-M_2_ = malvidin subgroup treated with higher dosage (18 mg/kg b.w.); Hypo-L/M_2_ = L-NIO (10 mg/kg b.w.) and higher dosage malvidin (18 mg/kg b.w.) subgroup.** Data are reported as Mean ± SEM; °*p* < 0.01 vs. Sham-saline subgroup, *p < 0.01 vs. Hypo subgroup.

Hypoperfusion and subsequent RE did not significantly affect eNOS and p-eNOS expressions both in cortex and striatum. eNOS and p-eNOS expression was increased in rats belonging to Hypo-M_2_ subgroup and blunted in rats of Hypo-L/M_2_ subgroup when compared to Hypo subgroup (Figures 2A,B). Moreover, the ratio p-eNOS/eNOS increased in Hypo-L/M_2_ subgroup, indicating an increase in expression and activity of p-eNOS in the malvidin-treated animals.

MMP-9 expression markedly increased in the cortex of hypoperfused rats; conversely, the MMP-9 expression significantly decreased in the cortex of animals treated with higher dosage malvidin compared to not-treated hypoperfused animals (*p* < 0.01 vs. Hypo subgroup). L-NIO did not interfere with the protective effects exerted by malvidin on the reduction in MMP-9 expression (*p* < 0.01 vs. Hypo subgroup; Figure 2C).

Gelatin zymography analysis did not show MMP-9 activity in sham-operated animals. After hypoperfusion/RE, MMP-9 activity was significantly increased in cortex and striatum of Hypo subgroup compared to Sham-saline subgroup. Conversely, MMP-9 activity was significantly reduced in rats treated with higher dosage malvidin compared to not-treated hypoperfused animals (*p* < 0.01 vs. Hypo subgroup).

**Figure 3:**
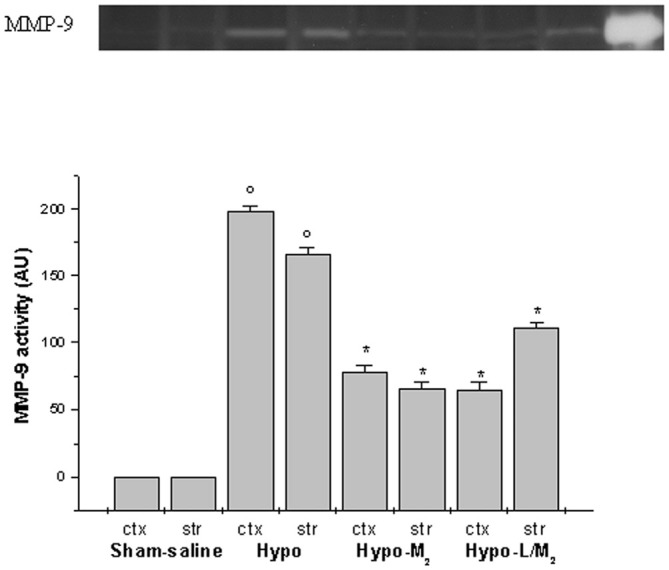
**MMP-9 activity detected by gel zimography assay, in two cerebral zones, cortex (ctx) and striatum (str), at the end of RE in Sham-saline = sham operated subgroup; Hypo = hypoperfused subgroup; Hypo-M_2_ = malvidin subgroup treated with higher dosage (18 mg/kg b.w.), Hypo-L/M_2_ = L-NIO (10 mg/kg b.w.) and higher dosage malvidin (18 mg/kg b.w.) subgroup.** Line 9: standard. Data are reported as Mean ± SEM; ° *p* < 0.01 vs. Sham-saline subgroup, *p < 0.01 vs. Hypo subgroup.

Moreover, L-NIO i.v. infused prior to higher dosage malvidin did not affect the protective effects of this anthocyanin on MMP-9 activity (Figure 3).

### Tissue Damage Evaluation and ROS Quantification

**Figure 4:**
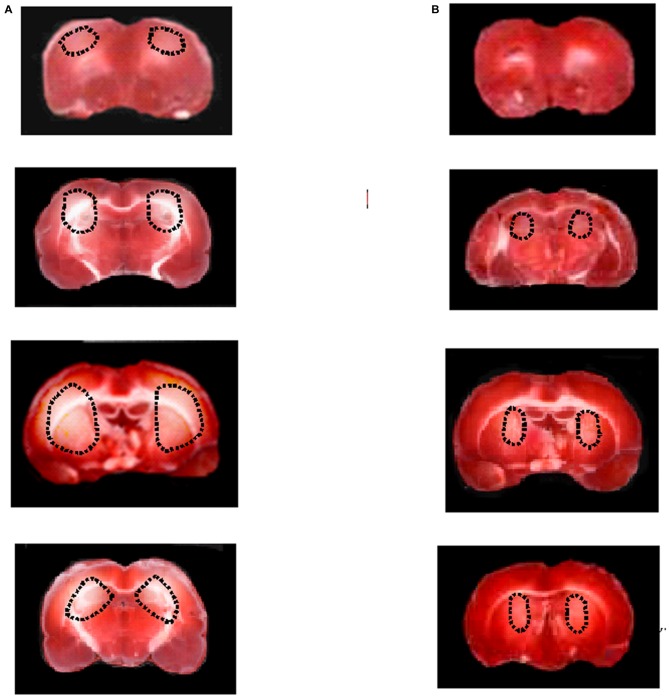
**Triphenyl tetrazolium chloride, (TTC) staining of coronal brain slices from a rat submitted to BCCAO and RE (A).** TTC staining of coronal brain slices from a rat treated with higher dose malvidin (18 mg/kg b.w.) **(B)**. The lesion in the striatum is outlined by the dashed black line.

The neuroprotective effects of malvidin were quantified by evaluating damaged area after 30 min BCCAO and 60 min RE. In Hypo subgroup, TTC staining showed marked lesions in cortex and in striatum in the both hemispheres (Figure 4A). However, hypoperfused rats treated with malvidin showed neuronal damage significantly reduced compared to animals belonging to Hypo subgroup (Figure 4B).

**Figure 5:**
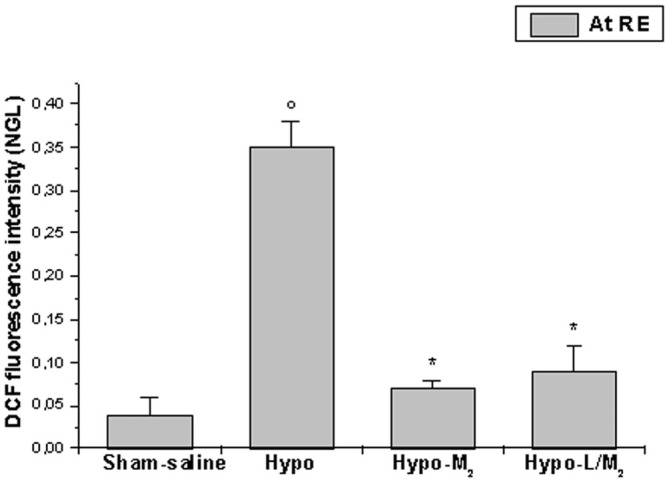
**DCF fluorescence intensity at the end of BCCAO and RE in the different experimental groups: Sham-saline = sham operated subgroup; Hypo = hypoperfused subgroup; Hypo-M_2_ = malvidin subgroup treated with higher dosage malvidin (18 mg/kg b.w.); Hypo-L/M_2_ = L-NIO (10 mg/kg b.w.) and malvidin (18 mg/kg b.w.) subgroup.** Data are reported as Mean ± SEM; °*p* < 0.01 vs. Sham-saline subgroup, *p < 0.01 vs. Hypo subgroup.

At the end of observations, DCFH-DA assay did not reveal significant increase in DCF fluorescence intensity in Sham-saline subgroup (0.04 ± 0.02 NGL). An increase in DCF fluorescence intensity was observed in Hypo subgroup after DCFH-DA superfusion, indicating marked ROS production (0.35 ± 0.03 NGL; *p* < 0.01 vs. Sham-saline subgroup). On the other hand, DCF fluorescence intensity was reduced in hypoperfused rats treated with malvidin compared to animals belonging to Hypo subgroup (Hypo-M_2_: 0.07 ± 0.01 NGL; *p* < 0.01 vs. Hypo subgroup). Finally, there was no difference in DCF fluorescence in rats treated with L-NIO prior to higher dosage malvidin administration, compared to animals belonging to Hypo-M_2_ subgroup (0.09 ± 0.03 NGL; *p* < 0.01 vs. Hypo subgroup; Figure 5).

## Discussion

The results of the present study indicate that 30 min BCCAO and subsequent 60 min RE caused marked alterations in the rat pial microcirculation, determining BBB disruption and significant neuronal damage. In hypoperfused animals, a significant decrease in arteriolar diameter, indeed, was accompanied by several microvascular impairments, including increase in venular fluorescent leakage and leukocyte adhesion, reduction in capillary perfusion and in capillary red blood cell velocity. Therefore, hypoperfusion by bilateral common carotid occlusion and RE were able to induce impaired vasomotor tone, BBB permeability and tissue perfusion reduction, triggering inflammatory mechanisms and affecting severely pial microcirculation, as previously reported (Lapi et al., 2012, 2015; Mastantuono et al., 2015). These results are in accord with the observations by Eklöf and Siesjö (1972); Eklöf and Siesjö (1973); who introduced this technique to investigate functional and structural impairment in cerebral circulation.

On the other hand, malvidin prevented, in dose-related manner, pial microvascular alterations, induced by hypoperfusion-RE, causing a significant increase in arteriolar diameter. This effect on arteriolar function was accompanied by prevention of vessel wall integrity, as demonstrated by the significant decrease in permeability, protection of capillary perfusion, as proven by improved PCL, and recovery of capillary red blood cell velocity, thus preventing marked inflammation. To clarify the molecular mechanisms triggered by the malvidin treatment, L-NIO, a specific eNOS inhibitor, was administered prior to malvidin, blunting the arteriolar dilation, but preserving the protective effects exerted by malvidin on the other vascular parameters. These findings allowed us to hypothesize a main role of eNOS in malvidin-induced preservation of arteriolar functions. Western blotting data support this hypothesis, demonstrating increased e-NOS and p-eNOS expressions in malvidin-treated rats. Moreover, the increase in the ratio *p*-eNOS/eNOS in the animals treated with malvidin indicates that there was an increase in expression and in activity of eNOS with the resultant arteriolar dilation. These results are in accord with previous studies carried out by Quintieri et al. (2013) who showed that malvidin is able to increase Akt/eNOS phosphorylation and to activate phosphatidylinositol-3-kinase (PI3-K)/NO/cGMP/PKG pathway in cardiac extracts, eliciting cardioprotection against ischemia/RE damage (Quintieri et al., 2013). Furthermore, our data indicate that malvidin treatment was able to counteract ROS production and neuronal loss, supporting the well known ROS scavenger effects of malvidin (Kähkönen and Heinonen, 2003). This molecule belonging to the family of anthocyanins, indeed, exhibits antioxidant, anti-inflammatory, anti-carcinogenic, anti-obesity and vasoprotective effects (Kähkönen and Heinonen, 2003; McGhie and Walton, 2007; He and Giusti, 2010). Therefore, these natural organic compounds may contribute to prevent the risk of chronic and degenerative diseases related to oxidative stress (Lapi et al., 2012, 2015; Lapi and Colantuoni, 2015; Mastantuono et al., 2015) and appear to be effective in preventing different pathophysiological conditions (Joseph et al., 1999, 2003). The present data support our previous observations that anthocyanins derived from Vaccinium Mirtyllus are able to counteract microvascular impairments, due to ischemia-RE in hamster cheek pouch model (Bertuglia et al., 1995).

However, the novelty in the present study is represented by the results on matrix metalloproteinase-9. This protelolytic enzyme has been suggested to induce deleterious effects during ischemia and RE injury. Lakhan et al. (2009) have shown that MMP-9 is released in response to ischemic insult from neurons, oligodendroglia, reactive astrocytes and activated microglia. Successively, Yamashita and Abe (2011) demonstrated that oxygen-derived free radicals, tissue-type plasminogen activator and other molecules are released after ischemic insult and could activate MMP-9 (Fagan et al., 2004; Yamashita and Abe, 2011). The increase in MMP-9 is further associated with different alterations, including excitotoxicity, neuronal damage (Lee et al., 2004), apoptosis (Lee and Lo, 2004; Copin et al., 2005), oxidative stress (Kelly et al., 2008), interference with oxidative DNA repair mechanisms (Yang et al., 2010) and BBB opening, leading to cerebral edema and hemorrhagic transformation (Zhao et al., 2006) after cerebral ischemia.

Our data demonstrate that MMP-9 levels increased in cortex after 30 min BCCAO and 60 min RE. This increase in MMP-9 expression was also accompanied by enhanced activity, both in cortex and striatum, as observed using gelatin zimography in hypoperfused animals. These data are in agreement with previous observations carried out by Fujimura et al. (1999), demonstrating increased MMP-9 expression and activity in a murine model of MCAO. On the contrary, malvidin-treatment appears to counteract MMP-9 expression and activity, significantly decreased in malvidin-treated rats, where we did not detect increased fluorescent leakage and interstitial edema.

The present data, according to our aims, demonstrate the effects of malvidin during cerebral hypoperfusion and RE injury utilizing a single molecule belonging to the anthocyanins. Therefore, for the first time, our results indicate that this natural antioxidant is able to reduce the expression of MMP-9 and the consequent increase in BBB permeability. During the last 20 years, MMP-9 has been identified as aberrantly overactive in ischemia, potentially causing deleterious effects during ischemia and after RE; therefore its inhibition has been suggested as a potential therapeutic target (Dong et al., 2009).

Clark et al. (1997) showed an increase in MMP-9 in human brain after ischemia. They reported that MMP-9 activity was markedly elevated in the infarcted human cerebral tissue after 2 days post-infarction when compared to non-infarcted tissue. Heo et al. (1999) used gelatin zymography and demonstrated that 2 h occlusion of middle cerebral artery caused transient increase in MMP-9, but did not affect MMP-2 activity.

Neuroprotection by MMP-9 inhibition or reduction in cerebral ischemia has been attempted in multiple studies during the last decade, using different strategies for MMP-9 inhibition. Under different pathophysiological conditions, MMP-9 inhibition has been proven to be neuroprotective.

Our results confirm that inhibition of MMP-9 may be useful in preventing cerebral edema, due to hypoperfusion and RE injury.

In conclusion, the malvidin’s effects may be related to the different mechanisms elicited by this anthocyanin: malvidin is able to stimulate NO release, causing arteriolar dilation, and to modulate eNOS, p-eNOS and MMP-9 expression. Consequently, malvidin is able to reduce inflammatory processes and to protect BBB integrity, contributing to the prevention of microvascular and tissue changes, triggered by brain hypoperfusion-RE injury.

## Author Contributions

DL, MC, MDiM, TM, LB, LS, SR, ADiC, NS, BG, MS, and AC conceived and designed the project, performed the experiments and the animal treatments, analyzed the data, wrote and reviewed the article.

## Conflict of Interest Statement

The authors declare that the research was conducted in the absence of any commercial or financial relationships that could be construed as a potential conflict of interest.
